# The Predictive Value of Prostate-Specific Antigen Density: A Retrospective Analysis of Likert 3 Multiparametric MRI of the Prostate

**DOI:** 10.7759/cureus.45782

**Published:** 2023-09-22

**Authors:** Oladapo Feyisetan, Victor Ezenwa, Mohammed Ramadhan, Merwi Al-Hadeyah, Olatunji Johnson, Jafar Hayat, Kingsley Ekwueme

**Affiliations:** 1 Urology, Leighton Hospital, Crewe, GBR; 2 Urology, Whiston Hospital, Whiston, GBR; 3 Medicine, Ministry of Health, Kuwait, Hawally, KWT; 4 School of Medical Sciences, The University of Manchester, Manchester, GBR; 5 Medicine, Ministry of Health, Kuwait, Kuwait City, KWT; 6 Statistics, The University of Manchester, Manchester, GBR; 7 Surgery, Ministry of Health, Kuwait, Kuwait City, KWT; 8 Urology, Glan Clwyd Hospital, Rhyl, GBR

**Keywords:** likert 3 prostate mri, psa density, pirads 3, multiparametric prostate mr, multiparametric mri (mpmri), pirads score, likert scale, prostate cancer

## Abstract

Background

Many international studies have covered the predictors of prostate cancer, but there is limited information pertaining to Likert 3 MRI scores and the diagnosis of clinically significant prostate cancer (cs-PCa). Therefore, this study aimed to assess the detection rate of significant prostate cancer in men with a Likert 3 score multiparametric MRI (mp-MRI) and the predictive value of prostate-specific antigen (PSA) density in detecting significant prostate cancer.

Methods

This is a retrospective analysis of patients referred for suspected confined prostate cancer. Inclusion criteria were patients with prostate mp-MRI score of Likert 3 and a prostate biopsy performed. Exclusion criteria included grossly abnormal feeling prostate, no biopsy performed, and an mp-MRI score (Prostate Imaging-Reporting and Data System/Likert) of 1, 2, 4, and 5. cs-PCa was defined as ≥ Gleason 3+4 prostate cancer. PSA density (PSAD) was calculated from MRI estimation of prostate volume. PSAD and histology results were subjected to receiver operating characteristic (ROC) curve analysis with the intention to assess the detection rate of significant prostate cancer in men with Likert 3 mp-MRI and the predictive value of PSAD in detecting significant prostate cancer.

Results

A total of 819 eligible men had a pre-biopsy mp-MRI scan taken between October 2019 and March 2022. A total of 177 men (21.6%, n = 819) were Likert 3 positive, and 31 did not proceed to take prostate biopsies. A total of 146 patients were included in the study. The median PSAD was 0.19 in men with cs-PCa. Prostate cancer was detected in 42 men (28.8% of the total included set), of which 27 (18.5%) had a Gleason 3+3 prostate cancer and 15 (10.3%) had Gleason ≥ 3+4 prostate cancer. Therefore, 35.7% (n = 42) of biopsy-positive men with Likert 3 mp-MRI had cs-PCa.

The ROC curve analysis confirms that PSAD is a predictor of cs-PCa. The optimal PSAD threshold was 0.16 (95% CI: 0.14-0.19), which gives an accuracy of 0.7371, a sensitivity of 0.7333, and a specificity of 0.7375.

Conclusion

The specificity of PSAD is arguably insufficient for it to stand alone as a decision-making tool when counseling men with equivocal mp-MRI on whether or not to undergo prostate biopsy. A predictive model will need to incorporate other independent risk factors. These may include lesion size, multiplicity, location of lesion(s), and age.

## Introduction

Prostate cancer is the second most commonly diagnosed cancer in men [1], with a lifetime risk in the UK being roughly one in six men [2,3]. As it is a common cause of morbidity and mortality, adequate screening is imperative to increase positive outcomes in patients. Prostate-specific antigen (PSA) is classically used as a screening tool but is associated with clinically insignificant findings and over-diagnosis [4]. Multiparametric MRI (mp-MRI) is a gold-standard tool that increases the probability of detecting clinically significant prostate cancer, avoiding unnecessary invasive procedures such as prostate biopsy [5-7].

A prostate MRI stratifies patients on a scale of 1 to 5 using Prostate Imaging-Reporting and Data System (PI-RADS, V2) [8], with a score of 1-2 indicating lower risk and 4-5 indicating a likely malignant lesion or clinically significant prostate cancer (cs-PCa) leading to a need for prostate biopsy [9]. A score of 3 is considered equivocal but patients currently undergo biopsy [10,11]. The Likert scale is also used and adheres to the PI-RADS V2 standardized structure, but adds clinical risk factors in addition to radiologist experience [8]. The Likert scale has been shown to outperform PI-RADS V2 to predict cs-PCa [12,13] and is recommended by the National Institute for Health and Care Excellence (NICE) in the UK [11].

The recommendation by NICE is supported by all prostate cancer and cs-PCa biopsy detection rates of 19-38.6% and 11-45%, respectively [14-18] within this group. On the flip side, it means that the majority of patients undergoing biopsies are either negative for prostate cancer or do not have cs-PCa. There is thus a need to build a reliable model aimed at predicting the presence of cs-PCa in men with a Likert 3 prostate MRI. At the moment, the increasing use of PSA density (PSAD) in combination with mp-MRI PI-RADS/Likert scores has been demonstrated to improve the prediction of cs-PCa in prostate biopsies and may further reduce the number of prostate biopsies performed [19-21]. Several studies support a PSAD threshold of ≥0.15 as an independent risk factor for cs-PCa [22-24], below which the risk of cs-PCa is low [25]. However, a few other studies support a lower PSAD threshold in combination with other independent risk factors, including age and MRI lesion characteristics (size, location, and multiplicity) prior to biopsy status. Increasing age > 68 years [23,24], lesions greater than 5 mm in diameter [15], multiple lesions [17], and lesions in the peripheral zone [23] were associated with an increased risk of cs-PCa. Prior negative prostate biopsy was associated with a reduced risk of cs-PCa [24]. We aimed to assess the detection rate of cs-PCa rate in men with equivocal (Likert 3) mp-MRI as well as the predictive value of PSAD in detecting cs-PCa prostate cancer to better determine men who should proceed to prostate biopsy and those who can safely avoid biopsy.

## Materials and methods

Patient population

This is a retrospective study. We performed a retrospective analysis of the hospital database at Glan Clwyd Hospital, Wales to identify men referred for suspected organ-confined prostate cancer between the dates of October 2019 and March 2022. We identified 819 men who had pre-biopsy mp-MRI of the prostate performed. Out of these, 642 (78.4%) men had mp-MRI scored at Likert 1, 2, 4, or 5 and were excluded. A total of 177 (21.6%) men had mp-MRI scored at Likert 3 and 31 men from this cohort did not have prostate biopsies and were also excluded. A total of 146 men were included in the study. The Likert scale was chosen as it adheres to most of the reporting structure in PI-RADS V2 with additional variables like radiologist experience and clinical risk factors. It is recommended in the UK by NICE for use in practice and has evidence supporting better detection of cs-PCa over PI-RADS scoring.

Prostate cancer

cs-PCa was defined as International Society of Urological Pathology (ISUP) group 2 (Gleason score 3+4) prostate cancer and above.

MRI

All the men had an mp-MRI of the prostate prior to biopsy with a 1.5T Siemens Sola machine (Siemens Healthineers, Erlangen, Germany). The protocol included high-resolution T2-weighted (T2WI) axial/coronal/sagittal, T1 axial wide field pelvis, T1 coronal wide field abdomen-pelvis, T2 axial wide field upper abdomen, diffusion-weighted (DWI) prostate, and dynamic contrast-enhanced (DCE) prostate. The MRI images were reviewed by three experienced uro-radiologists. PSAD was calculated from MRI estimation of prostate volume and included in the report.

Prostate biopsy

Transrectal ultrasound (TRUS)-guided biopsies were performed if the lesions were large or multiple and located in the peripheral zone on mp-MRI. Five systematic biopsies were taken from each lobe of the prostate.

Transperineal (TP) biopsies under TRUS guidance were performed if the lesion was small and solitary or located in the anterior zone. Targeted biopsies of the lesion as well as systematic biopsies from each lobe were taken. Biopsy samples were labeled to indicate the region of the prostate sampled, and reported accordingly.

Images and pathology were reviewed at a multidisciplinary meeting with reporting uro-radiologists, pathologists, and urologists in attendance.

Statistical analysis

The factor evaluated as a predictor of cs-PCa was PSAD. To determine the optimal PSAD threshold for detecting men with cs-PCa, a receiver operating characteristic (ROC) analysis was conducted using the R statistical software (version 4.05; R Foundation for Statistical Computing, Vienna, Austria) and the cut-off point package [26]. The threshold was determined through the maximization of the Youden index [26], which is a measure of the accuracy of a binary classifier calculated as the difference between sensitivity and false positive rate. To estimate the uncertainty of the threshold, a bootstrap resampling technique was applied with 1000 repetitions.

## Results

Patient characteristics

The median age, PSA, and prostate volume as well as the range of each variable for our patient cohort (Table 1) was 65 years (46-84), 5.6 ng/ml (1.1-32), and 53 ml (17-220), respectively. Overall, the median PSAD was 0.11, ranging from 0.02 to 0.65. Median PSAD in men with cs-PCa was 0.19, ranging from 0.08 to 0.46.

**Table 1:** Patient characteristics PSA: prostate-specific antigen.

Characteristics (median and range)	Value
Age	65 years (46-84)
PSA	5.6 ng/ml (1.1-32)
Prostate volume	53 ml (17-220)
PSA density	0.11 (0.02-0.65)

MRI

All men had the same mp-MRI protocol performed on a 1.5T Siemens Sola machine. We stratified patients with respect to their Likert scores below (Table 2).

**Table 2:** Stratification of patients with their respective Likert scores

Likert score	Number of men
1	2
2	287
3	177
4	112
5	241
Total	819

Biopsy technique

A total of 146 men had prostate biopsies, of which 118 (80.8%) had TRUS biopsies while 28 (19.2%) had TP ultrasound biopsies performed.

Biopsy histology

Table 3 details our biopsy types for histology. Prostate cancer was detected in 42 (28.8%) of the 146 men in the study, of which 27 (18.5%) had Gleason 3+3 (ISUP group 1) prostate cancer and 15 (10.3%) had Gleason ≥ 3+4 (ISUP group 2 and above) prostate cancer (Table 4). Histology results were then matched to the PSAD of our cohort (Table 5).

**Table 3:** Biopsy outcomes in relation to biopsy type

Type of biopsy	n (%)
Transrectal	118 (80.8%)
Transperineal	28 (19.2%)

**Table 4:** Biopsy outcomes regarding histology results All prostate cancer (28.8%) was broken down into Gleason 3+3 and significant prostate cancer (18.5% and 10.3%), respectively. Significant prostate cancer, as we defined in our study, was further broken down into Gleason 3+4 and above to a total of 10.3%.

Histology results	n (%)
All prostate cancer	42 (28.8%)
Gleason 3+3 prostate cancer	27 (18.5%)
Significant prostate cancer	15 (10.3%)
Gleason 3+4	8 (5.5%)
Gleason 4+3	5 (3.4%)
Gleason 8	1 (0.68%)
Gleason 9	1 (0.68%)
No prostate cancer	104 (71.2%)

**Table 5:** Biopsy outcomes: histology results matched to PSA density PSA: prostate-specific antigen.

Gleason score	Number of cores involved	Overall sample involvement (%)	Maximal core involvement (%)	PSA density
3+4	2	5	10	0.05
3+4	1	15	100	0.08
4+3	1	1	5.6	0.1
3+4	4	25	18	0.11
3+4	2	2.5	9	0.14
8	4	25	100	0.16
4+3	5	30	58	0.18
3+4	2	1	5	0.19
4+3	5	4	20	0.19
3+4	2	5	20	0.2
4+3	4	10	40	0.2
9	2	1	8	0.2
4+3	4	10	35	0.21
3+4	1	2	11	0.23
3+4	4	80	88	0.46

PSAD evaluation and prediction of cs-PCa

**Figure 1:**
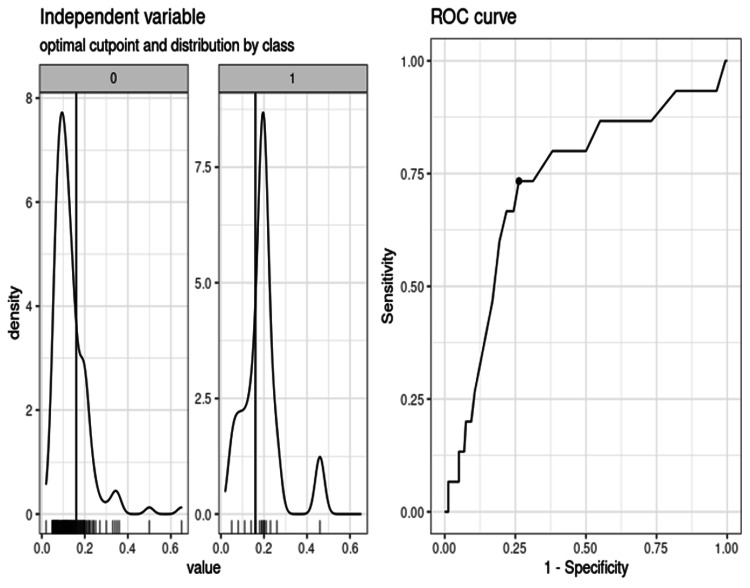
ROC curve demonstrating the optimal PSA density threshold for predicting cs-PCa PSA: prostate-specific antigen; ROC: receiver operating characteristic; cs-PCa: clinically significant prostate cancer.

The optimal threshold for identifying cs-PCa using PSAD was found to be 0.16 (95% CI: 0.14-0.19). This threshold gives an accuracy of 0.7371, a sensitivity of 0.7333, and a specificity of 0.7375, as seen below (Figure 1).

## Discussion

Multiparametric MRI demonstrates very good predictability at the polar ends of the PI-RADS scale with a negative predictive value of 63-98% for high-risk prostate cancer [5,7].

The Likert scale incorporates clinical risk factors as well as the radiologists’ experience and outperforms the PI-RADS scale [12,13]. Whichever reporting scale is used, the equivocal Likert 3 or PI-RADS 3, imaging remains a challenge. The proportion of mp-MRI scans reported as equivocal could be considered a surrogate marker of the MRI machine, imaging protocol, and radiologist experience. This study found that 21.6% of the total mp-MRI images were reported as Likert 3 prostate cancer; such a rate is comparable to other studies estimating 19-38.6% [7,14,16].

It is also important to note that the algorithm will aid patient-physician decision-making when the MRI is equivocal, but will not deny an informed man the choice to proceed with a prostate biopsy.

The overall prostate cancer detection rate was 28.8% and cs-PCa cancer was detected in 10.3% of the men with Likert 3. Within this subgroup of biopsy-positive men, 35.7% had clinically significant prostate cancer while 64.3% had indolent prostate cancer. Again, these results are similar to previous studies estimating a rate of 11-45% [14-18].

PSAD was a predictor of cs-PCa from the area under the curve (AUC) analysis with a sensitivity of 0.73 and a specificity of 0.74 at an optimized PSAD threshold of 0.16. This threshold is similar to several studies [22,23,25,27]. However, using this threshold would have missed four out of 15 cs-PCa if no biopsy was performed. This is a miss rate of 26.6%, even higher than a 10% potential miss rate reported in other studies [23]. Adjusting the PSAD threshold to 0.15 made no difference to the miss rate in this study. Lowering the PSAD threshold further would sacrifice specificity and increase the number of men referred for prostate biopsy as well as the detection of indolent prostate cancer.

Limitations

The limitations of this study include but are not limited to the fact that retrospective analysis of data lends itself to bias, where results could change in prospective follow-up. A relatively small sample size is within this study, as it is expected that the population of mp-MRI scored as Likert 3 will be small when reported by experienced uro-radiologists. Our study was done in a single center with multiple clinicians performing prostate biopsies. The percentage of Gleason 4 in patients in ISUP group 2 was unfortunately not assessed in this study, as that would complicate analysis.

There was no option to perform fusion biopsies in our hospital. We appreciate MRI/TRUS-fusion targeted biopsies have a higher diagnostic yield compared with systematic TRUS-guided biopsies. However, studies comparing cognitively targeted TRUS biopsies with fusion biopsies have yielded mixed results.

We also utilized 1.5T MRI images. 3T mp-MRI machines do produce better images, but we found no evidence that the reporting outcomes from 1.5T mp-MRI are inferior to 3T MRI machines in mp-MRI of the prostate, therefore warranting no significant concern. Various nomograms have been proposed, combining multiple clinical parameters, while we only studied PSA. We agree with this sentiment, but none of the proposed nomograms have been validated and widely agreed upon either nationally or internationally. As authors, we chose PSAD because this is currently the most widely used “objective” surrogate marker in deciding if a patient with an mp-MRI with a score of Likert 3 or PI-RADS 3 should proceed to prostate biopsy (see NICE UK recommendation). This study contributes to the body of evidence that PSAD alone is inadequate.

## Conclusions

The use of mp-MRI has lived up to the claims of the PROMIS study, and 35% of men in this study safely avoided prostate biopsy. The use of PSAD, calculated from mp-MRI, has gained traction as a reasonably predictive decision-making tool when counseling men with equivocal mp-MRI on whether or not to undergo prostate biopsy. However, this study has demonstrated that the specificity of PSAD with regards to cs-PCA in an equivocal mp-MRI is arguably insufficient for it to be a lone surrogate factor in reaching a decision on prostate biopsy as a stand-alone investigation. There is still a need for a better predictive model to guide the recommendation of prostate biopsy for men who underwent an mp-MRI and while PSAD will certainly be a significant variable in determining this improved predictive model, other independent factors are required and may be implemented in the future. These may include lesion size, number of lesions, location of lesion(s), and age.
